# The Role of New Technological Opportunities and the Need to Evaluate the Activities Performed in the Prevention of Diabetic Foot with Exercise Therapy

**DOI:** 10.3390/medicines8120076

**Published:** 2021-12-02

**Authors:** Piergiorgio Francia, Alessandra De Bellis, Giulia Iannone, Rosy Sinopoli, Leonardo Bocchi, Roberto Anichini

**Affiliations:** 1 Diabetes Unit and Diabetes Foot Unit, General Hospital of Pistoia, 51100 Pistoia, Italy; roberto.anichini@uslcentro.toscana.it; 2 Diabetes and Metabolic Diseases Unit, San Giovanni di Dio Hospital, 50143 Florence, Italy; alessandra.debellis@uslcentro.toscana.it; 3 Department of Information Engineering, University of Florence, 50124 Florence, Italy; giulia.iannone@virgilio.it (G.I.); rosy.sinopoli@stud.unifi.it (R.S.); leonardo.bocchi@unifi.it (L.B.)

**Keywords:** diabetes, foot ulcer, physical activity, exercise, technologies, proper execution

## Abstract

The diabetic foot (DF) is one of the most feared conditions among chronic complications of diabetes, which affects a growing number of patients. Although exercise therapy (ET) has always been considered a pillar in the treatment of patients at risk of DF it is not usually used. Several causes can contribute to hindering both the organization of ET protocols for Diabetes Units and the participation in ET programs for patients at different levels of risk of foot ulceration. The risk of favoring the occurrence of ulcers and the absence of clear evidence on the role played by ET in the prevention of ulcers could be considered among the most important causes leading to the low application of ET. The increased availability of new technologies and in particular of systems and devices equipped with sensors can enable the remote monitoring and management of physical activity performed by patients. Consequently, they can become an opportunity for introducing the systematic use of ET for the treatment of patients at risk. Considering the complexity of the clinical conditions that patients at risk or with diabetic foot ulcer can show, the evaluation of how patients perform the ET proposed can consequently be very important. All this can contribute to improving the treatment of patients and avoiding possible adverse effects. The aim of this brief review was to describe that the use of new technologies and the assessment of the execution of the ET proposed allows an important step forward in the management of patients at risk.

## 1. Introduction

Diabetic foot (DF) is a long-term diabetes complication that can increase morbidity and mortality in addition to affecting mobility and the overall well-being of patients. In particular, the DF has a complex multifactorial pathogenesis that makes it difficult to prevent and treat [1]. In this sense, it is well known that the prevention and treatment of DF disease requires a multidisciplinary approach.

Patients with diabetes have a high lifetime risk of developing DF, with an annual incidence of around 2% [1,2]. In two thirds of cases there is a re-ulceration in the following three years even in the case of healing and it has been reported that up to 85% of diabetes amputations are preceded by a foot ulcer [3,4,5,6]. Due to the presence of multiple complications, the five-year mortality rate among patients with diabetes who have undergone major amputation procedures was reported to be higher than 40% [7,8,9]. Most patients with DF disease exhibit a similar path to ulceration. Ulcers often result from a person with diabetes having concurrent or multiple risk factors where diabetic peripheral neuropathy and peripheral artery disease usually playing a key role. In addition, more than 50% of DF ulcers become infected and about a fifth of these sufferers undergo amputation [1,6,10]. These epidemiological data show that DF represents a serious health problem in many countries of the world. Thus, it is necessary to continue improving the treatment offered to patients.

Even though the importance of exercise training (ET) has been recognized for about a century, the involvement of patients in a specific exercise training protocol is still not systematic [11,12,13]. Teams of multidisciplinary specialists do not use ET in the treatment of patients at risk for many reasons [5,12,14].

Movement, and in particular walking, or other activities that involve stress (e.g., plantar pressure) are considered risk factors for DF [6,13,15]. Furthermore, there is still no definitive evidence that the protocols proposed have a significant preventive effect [13,16,17].

Further limitations in adopting and participating in ET protocols can occur both for diabetes services and patients themselves. The former may not have the necessary resources to organise ET programs (i.e., space, materials, personnel), while the latter who show different personal and health conditions may not be able to participate in the ET program [5,18].

Involving patients at risk in ET courses requires that they be appropriately tailored to them. Furthermore, their regular and consistent participation in the ET activities is very important. This is because a number of positive results obtained through ET can be lost once the training ceases, and needless stress could be caused by the interruption, both on metabolism and on body structures [18,19,20,21].

Nowadays, we are experiencing a transition period characterized by strong technological evolution that has deeply revolutionized the healthcare system as well as society in general. The new phase we entered several years ago has been accelerated due to the pandemic caused by the coronavirus disease (COVID-19). This has resulted in strong digitalization of services and the diffusion of telemedicine and smart health [22,23,24,25]. In this context, technology and digitalization could bring increasingly more important changes to the management of the relationship between subjects at risk and ET. This could also intensify the ET program: the increasing availability and wider use of technological solutions enables the multiparametric monitoring of movement, as well as of the health conditions of patients. Consequently, digitalization can represent an opportunity to encourage patients at risk to participate in the ET program [26,27,28,29].

To date and to the best of our knowledge, there are no studies that have assessed the quality of the execution of ET protocols performed by individual patients. The assessment of the quality of execution of the prescribed activities could ensure the achievement by patients of the objectives of the ET programs, and it would reduce the risks associated with participation.

The aim of this article was to describe how the use of new technologies and the systematic assessment of the quality of the ET protocol performed by patients can be an important step forward in the management of the relationship between patients at risk and ET.

## 2. Exercise Training and Diabetic Foot

Numerous studies have reported that periods of ET can lead to a significant improvement of important ulcerative risk factors [20,30,31,32,33,34].

However, multiple factors must be considered in the organization of ET programs for the prevention of DF. In addition to the level of risk for ulcer and more in general the whole condition of each patient, several other personal factors that can limit the participation to the ET programmes should be considered [5,18,21].

In patients with diabetes, it is well known that the presence of peripheral neuropathy (PN) and therefore the loss of protective sensation (LOPS) can reduce the sensitivity of the foot and the ability to perceive harmful events such as excessive plantar pressure and stress that may occur during the ET session [5,6,35,36]. Furthermore, the presence of PN can lead to the progressive development of further feared ulcerative risk factors such as alterations in balance, posture, muscle strength and joint mobility, all factors that can negatively affect the biomechanics of gait [5,20,37]. The presence of peripheral vasculopathy can further increase the ulcerative risk and negatively affect the daily physical activity (PA) resulting in pain at rest, claudication and reduced walking speed and distance. These factors, in addition to other conditions (i.e., foot deformities and calluses) that can contribute to the onset of ulcers following walking should be promptly identified before intervening on the lifestyle of patients at risk [21,37,38,39].

While previously ET protocol studies usually addressed patients with very low, low and moderate risk of ulceration, recent studies have instead aimed to study patients at higher risk (i.e., with an ongoing ulcer or one that has healed in the previous months). In the management of patients with high risk of ulcer, movement is usually severely restricted. For preventing and healing foot ulceration, one of the main goals is to reduce high foot pressure also with the use of offloading devices [6,40,41,42]. As proposed by recent studies, for these patients the early introduction of ET protocols may be important also to avoid over protecting the foot and the possible negative consequences associated with that. Early intervention could avoid further compromising several parameters such as muscle strength, joint mobility, balance and walking quality as well as vascularization and sensitivity, which are already strongly affected in these patients [35,40,41,43,44].

Supervised treatment has many advantages such as the fact that the therapist can give timely instructions, show and explain correct execution as well as guiding and motivating the same execution. It can also at the same time prevent ulcers by avoiding behaviours or situations that could risk ulcers [13,45].

However, two aspects must be considered that Diabetes Units can get into evident difficulties in organizing a regular service for the treatment of patients at risk through ET protocols and the necessary participation of patients at these activities can be hindered by several factors. It should be considered that personal and environmental conditions (e.g., poor health, long trips, time constraints) may limit the participation of patients in ET protocols. In this sense, the evaluation of aspects such as lifestyle and living environment as well as the same network of patients’ social relationships can now be more and better studied thanks to the use of new technologies [5,46]. Among the objectives pursued by ET protocols that, in turn, are addressed to patients at risk or with DF ulcers there is the improvement of: join mobility, balance, quality and control of posture, quality of gait, muscle strength, peripheral vascular and nerve function, metabolic control, plantar pressure distribution and ulcer healing [13,21,30,32,40]. The aim of one of the first studies [19] investigating the relationship between ET and the prevention of foot ulcer was the improvement of joint mobility [19].

Exercises aimed at increasing joint mobility of the ankle and foot paying specific attention to the toes, are considered a cornerstone in the treatment of patients at risk or with DF ulcer [21,31,34]. Joint mobility can be improved by performing active and passive joint mobilization. These exercises can be performed in weight-bearing and non-weight- bearing condition and due to the adoption of different postures (i.e., standing, sitting and lying supine). Specifically, the exercises in non-weight-bearing condition are regularly used in the treatment of patients with ulcer in order to improve healing [13,16,33]. In these patients, for the purposes of reducing the weight-bearing activities different solutions of equipment such as the mini-trampoline can be used for improving joint mobility [34]. Exercises aimed at improving joint mobility and flexibility should be addressed to the whole body paying specific attention to the structure of the trunk as well as the proximal ones of the lower limb (i.e., thigh and pelvis). In fact, over the years, the significant postural and biomechanical alteration that patients at risk may show, can affect the condition of other proximal structures of the body [21,33].

Numerous protocols propose exercises aimed at improving static and dynamic balance, proprioceptive sensitivity as well as walking skills and foot rollover [20,31,33,34]. In this regard, it has been shown that these objectives can also be pursued by performing exercises aimed at improving the static and dynamic posture [21]. Generally, these exercises aim at improving plantar pressure distribution and reducing the risk of foot ulcer itself [33,35]. 

The improvement of muscle strength in addition to muscle coordination and metabolism represents an important further goal in the treatment through ET of both patients at risk and those with ongoing ulcer [31,33]. In this sense, an ET program can interrupt the vicious circle that involves significant muscle reduction particularly evident in the lower limbs. Moreover, using equipment or performing body weight exercises also in the short term (e.g., 12 weeks) it is possible to significantly increase the muscle strength of the lower limbs [21,44]. Considering the setting of ET, circuit training which includes strengthening exercises can improve muscle strength in addition to gait speed, balance and walking skills [20].

The ET protocols can include a warm-up and cool-down phases aimed at avoiding injuries and promoting the correct execution of the planned exercises. During these initial and final activities, different exercises such as various types of activities involving walking, cyclette, arm ergometry, and flexibility can be included [31,34].

A number of studies have foreseen different phases with progressively more demanding protocols. Patients were able to move on progressively to the following phase after the correct execution of the activities assigned had been assessed [31,33,34].

## 3. Remotely Supervised Exercise Intervention: A Closed Loop System

Nowadays, technological solutions are available that can allow to define a complex system for monitoring and managing patients at risk who are involved in physical activity. In particular, it is also possible to monitor in real time and remotely more and more factors through the use of sensors during an ET session. This could allow therapists to supervise the training sessions performed by patients even if they are not present (e.g., Microsoft Kinect device; ExerciseCheck) [22,23,24].

In addition to monitoring the condition of patients, the environments and equipment used during the sessions can also be agreed upon in advance and supervised during the sessions. Pressure sensors are among the most used wearable devices in the prevention of diabetic foot (e.g., Orpyx Medical Technologies Inc., Calgary, AB, Canada; Medixfeet Insole®, Thorsis Technologies GmbH, Magdeburg, Germany) [47,48].

These types of sensors are easily readable, user-friendly and can be placed on different supports such as at the level of the insoles and smart socks. The evaluation of the pressures exerted at the level of the foot has been carried out for over half a century and it is increasingly used [49,50,51]. Indeed, the possibility of assessing the pressures exerted on the patient’s foot even during ET is of great importance in the prevention of ulcers especially in patients with loss of protective sensation or those patients with lesser or absent ability to perceive harmful events such as excessive plantar pressure and stress [35,36,52].

Movement sensors such as accelerometers are also considered very important and have been widely used in the study and treatment of patients at risk [18,27,43,53]. Thanks to the use of movement sensors (e.g., ActiGraph wG3TX-BT, Pensacola, FL, USA; Sensoria socks, Sensoria Inc., Redmond, WA, USA) it is possible to collect data on the intensity, duration and type of activity performed. Furthermore, a number of devices can also provide information on posture (lying, sitting, orthostatic or dynamic position) and even on the same quality of gait also thanks to the use of different devices and video analysis (BioStampRc from MC10, Lexington, MA, USA). All this opens up new opportunities for a preliminary analysis of gait by remote [27,54,55,56,57,58]. Many of these devices used for monitoring one or more parameters simultaneously are equipped with wireless sensors and easily connectable to Internet. This can be particularly useful in the remote supervision of patients at risk during ET [28,29,51,53].

Other sensors in addition to those aimed at measuring movement and its effects can be used for remote monitoring providing important information [29,53]. Image acquisition plays another important role for the treatment of patients at risk. Videos could also be used for a summary evaluation of gait (e.g., Smart Prevent Diabetic Feet Application –SPDFA; Microsoft Kinect device) [22,59,60,61]. Sensors detecting temperature, foot humidity and galvanic skin response have been used in the monitoring of patients at risk (e.g., Siren, washable smart socks, San Francisco, California; Medixfeet Insole®; Thorsis Technologies GmbH, Magdeburg, Germany; Podimetrics SmartMatt, Somerville, Massachusetts) [22,53,59,60,62]. As proposed by previous studies, even devices for the monitoring of physiological parameters such as glycaemia and insulin therapy or variables such as climate and environmental conditions (i.e., geolocation) can be used (e.g., Body Cardio Scale; Nokia, Helsinki, Finland) [43,53,59,63].

Such accurate remote monitoring is promoted by the availability of mobile network and the ever-increasing diffusion worldwide of the Internet of Things. Moreover, it is nowadays possible to set up systems allowing communication between patients and diabetes centers thanks to these technological advances [23,26,64]. The flow of information generated by these devices can be managed and processed locally through the use of different types of devices including mobile phones and tablets or this flow can be transmitted to centralized units such as diabetes centers. Flows of information both peripherally and centrally can also be used in real time to monitor, analyze and guide the activity of patients themselves (e.g., Smart Prevent Diabetic Feet Application -SPDFA) [22]. Thanks to outgoing means of communication (i.e., alarms, messages and calls), the same central units can communicate with patients, with the devices they used or they can manage the spaces in which the activities are being performed [50,51,65]. These outputs may involve changes in the environment or in the behavior of patients themselves and generate feedback. Nowadays, the technological resources easily available even at low costs, can create a closed-loop type system which, although with many limitations, can represent an important response to the need of monitoring patients at risk who have participated in an ET session.

## 4. Monitoring and Evaluation of Proper Execution of Exercise

Studies aimed at verifying the effect of ET programs in the prevention of DF, did not commonly assess how the exercises were performed [31,45]. Although the aim of these studies was to verify the effect of an ET protocol it may be important to define the quality of the execution of the protocol itself [19,20,32].

This could be important in order to better understand both the effectiveness of the treatment proposed and which exercise or activity is the most useful. The correct execution of every activity within a protocol of an ET program cannot always be taken for granted.

This is why patients at risk may show impairments affecting the musculoskeletal system and other systems due to diabetes and/or other pathologies that could hinder the correct execution of the ET protocol. Therefore, the differences among patients (e.g., age, comorbidities, training status) mean that part of the exercises or the activities proposed can be difficult to perform by patients themselves. Consequently, it is of prime importance to consider the difficulties in performing exercises. Indeed, these problems in terms of the execution of exercises, that in a number of cases can be very evident, can make the ET less effective and therefore be a risk for the patients themselves [31,45].

The complexity of the condition of the patients at risk can make it necessary to adapt the training protocol especially during the first months of activity. The same significant improvements in muscle strength, joint mobility, walking speed and other important parameters may require the proposed ET to be adapted several times. All this can be possible only where the system of continuous evaluation and monitoring of patients is carried out.

## 5. Discussion

In this context, the advances in technology of the health service could finally allow the development of a relationship that was never really achieved. The massive introduction of technological solutions in the field of healthcare can allow the rethinking of the relationship itself between exercise and the prevention of diabetic foot. An important result of using technological solutions for monitoring, even remotely, patients at risk during an ET session, may bring the involvement of a higher number of patients as well as promoting their regular participation. Reducing the use of supervised activities in favour of those unsupervised or partially supervised can be another possible result linked to the remote monitoring of patients during ET [31,66].

However, the monitoring of the patient by remote during the ET sessions may be limited affecting also the quality of the execution of the exercise protocol itself. This suggests it is useful to maintain the possibility for patients with particular conditions to be able to perform the ET protocol in the presence of a therapist when needed. It may also be important to organize a counseling service within the diabetes centers, which can be contacted remotely and that provides support for patients who participate in an ET program or who, more generally, practice physical activity (PA).

The implementation of an appropriate monitoring system can allow the creation of a closed-loop system in which the input information obtained from the monitoring itself is stored, analyzed and used to provide output. The aim of this system would be to monitor a large number of patient-related or environmental parameters and then intervene through various types of outputs on the behavior of the subjects or on all the parameters monitored.

In this sense, multiparametric information on patients and the environment can be transmitted, collected and processed with increasingly different systems such as Data Mining, Artificial Intelligence and therefore Machine Learning and Deep Learning [63,67,68,69,70]. The use of these resources can be useful in extracting more and more knowledge from the information collected. Moreover, this information can allow the definition of PA that the patient will have to perform or avoid.

All this would be aimed at guaranteeing the correct execution of the ET protocol and avoiding injuries to patients themselves. However, the real possibility of adopting an ET protocol in the treatment of patients at risk depends on its real role in the prevention of DF and how it positively affects the health condition of high-risk subjects or of those with ongoing ulcers. In this article, we have suggested the evaluation of the execution of the protocols proposed as added value to the increasing use of ET in the treatment of patients at risk. Indeed, still today, the method of execution of individual parts of the assigned protocols or of the whole protocol itself is not evaluated and, this can represent a limit in the definition of increasingly effective protocols and sometimes in guaranteeing the safety of patients themselves. There are many factors that can hinder the correct execution of the ET.

## 6. Conclusions

The risk of the occurrence of a foot ulcer, the lack of definitive evidences on the real role of ET in the prevention of DF as well as the difficulty of organizing and participating in ET programs are among the most important limitations that have hindered the relationship between PA management and DF prevention strategy. Nowadays, the availability of increasingly advanced technological solutions can finally allow the systematic use of ET in the prevention of DF and in the treatment of patients with active ulcers.

In this sense, the multiparametric monitoring of patients and their living environment in addition to the transmission and processing of the collected data can allow the appropriate management of patients at risk during the ET sessions. The set of indications that diabetes centers can send to patients and that have been drawn up considering the information collected by the monitoring can allow the creation of a closed loop system for the management of patients who practice physical activity. Finally, the evaluation of the quality of the execution of each activity included in the ET program can be a further important step towards making the ET a key element in the treatment of patients at risk.
